# Building India’s Low-Carbon Healthcare System: A Comprehensive Review of National Policies, Operational Frameworks, and Decarbonization Strategies

**DOI:** 10.5334/aogh.5096

**Published:** 2026-01-28

**Authors:** Harshini Thirumoorthi, Saravanan Sampoornam Pape Reddy

**Affiliations:** 1 Arunai Medical College & Hospital, Tiruvannamalai, Tamil Nadu, India; 2 Department of Periodontology, Army Dental Corps, New Delhi, India

**Keywords:** climate change, healthcare sustainability, decarbonization, climate-resilient health systems, India, health policy, green hospitals

## Abstract

*Background:* Climate change poses a profound threat to public health globally and in India, but ironically healthcare activities themselves contribute significantly to greenhouse gas emissions. The healthcare sector produces about 4–5% of global carbon emissions. India’s healthcare system is estimated to be the world’s seventh-largest carbon emitter in absolute terms. To align with climate goals and protect health, India has begun instituting policies to foster a low-carbon, climate-resilient healthcare system.

*Objective:* To comprehensively review India’s national policies, operational frameworks, and strategies aimed at reducing healthcare-related carbon emissions (decarbonization) while strengthening health system sustainability.

*Methods:* We conducted a narrative review of policy documents and PubMed-indexed literature from 2018 to 2025 focusing on climate change mitigation in the Indian health sector.

*Findings:* India has mainstreamed health into national climate planning with the release of the National Action Plan on Climate Change and Human Health (2018) and the launch of the National Programme on Climate Change and Human Health (2019). These set targets for climate–health surveillance and low-carbon, climate-resilient medical health facilities. Climate action and sustainability are being included in accreditation standards. Early benefits include: greater energy reliability and lower cost energy services for healthcare centers with solar energy and higher health facility performance during extreme weather events. However, implementation remains nascent. Gaps remain in data, funding, and capacity to scale low-carbon practices across India’s sprawling health system.

*Conclusions:* India has made initial strides in aligning its health sector with climate mitigation imperatives through policies and guidelines for sustainable, low-carbon healthcare. Strengthening governance, financing, and technical support will be critical to fully implement decarbonization strategies. A low-carbon healthcare system in India can not only reduce emissions but also improve public health, resilience, and healthcare quality, setting an example for sustainable healthcare development in low- and middle-income countries.

## Introduction

Climate change is widely recognized as an urgent public health challenge. Rising temperatures, extreme weather events, shifting disease patterns, and air pollution are already impacting health in India [1]. Paradoxically, the healthcare sector, meant to safeguard health, is itself a significant source of greenhouse gas (GHG) emissions. Globally, healthcare operations account for an estimated 4.4% of net GHG emissions. This carbon footprint of healthcare is comparable to that of major industrial sectors, and if the global health sector were a country, it would rank among the top emitters [2]. India’s healthcare system is a substantial contributor: in absolute terms, it has been estimated as the seventh-largest national healthcare emitter. While exact figures vary by estimation method, healthcare-related emissions likely constitute on the order of a few percent of India’s total GHG emissions [3]. Major sources include hospital energy use (often from coal-based electricity), transport (patient and supply chain logistics), and the production of medical supplies and pharmaceuticals. For instance, energy-intensive hospital units, such as operating theaters and intensive care units, are among the largest emitters within healthcare facilities. This dual role of healthcare, vulnerable to climate impacts yet contributing to the problem, creates a compelling imperative to transition toward sustainable, low-carbon healthcare systems [4].

Establishing a low-carbon healthcare delivery system is especially important in India. India is particularly susceptible to the effects of climate change on health. Heat waves have become more frequent, longer lasting, and more severe in the past two decades, and their emergence has been associated with increased deaths, heat-related illnesses, and healthcare services demand. Concurrently, climate change-induced extreme weather events (e.g., floods and cyclones) are increasing in severity, disrupting health infrastructure, destroying healthcare facilities, preventing service provision, and escalating health risks to the most vulnerable [5]. But at the same time, India has made ambitious national climate pledges under the Paris Agreement and committed to reaching net-zero emissions by 2070 [6]. In parallel, health sector contributions to mitigation can have major co-benefits. Lowering healthcare emissions will not only help India reach climate targets but it would also improve air quality (and thereby health), save money through energy efficiency, and make the health system more resilient (such as solar-powered facilities that can run during grid outages or disasters). Further, connecting to climate sustainability grounds health equity in the ethical notion of “first, do no harm,” so that care delivery does not systematically harm public health through environmental pollution [7].

Recognizing these imperatives, the Indian government has started integrating climate change into health planning and policy. In 2015, India’s National Action Plan on Climate Change (NAPCC) was expanded to include a dedicated mission. This culminated in the development of the National Action Plan on Climate Change and Human Health (NAPCCHH) in 2018, a strategic framework to address climate-related health risks and promote sustainable practices in healthcare [8]. In 2019, the Ministry of Health and Family Welfare operationalized these ideas by launching the National Programme on Climate Change and Human Health (NPCCHH) under the National Health. The NPCCHH marked a significant step: it identified priority areas (such as mitigating health impacts of heat waves and air pollution) and explicitly called for “green and climate-resilient” healthcare facilities across India. Since then, a range of policies and operational frameworks have emerged from green building guidelines for hospitals to renewable energy initiatives signaling a nascent but growing movement toward low-carbon healthcare [9].

In this article, we offer a review of India’s strategies for creating a low-carbon healthcare system that is sustainable. We explore the national policies, programmatic frameworks, and on-the-ground interventions through which healthcare can be decarbonized as it is made healthier. We concentrate on events occurring since 2018, as this has marked the uptick in attention to climate and health in India. In this review, we aim to update public health researchers on the status, opportunities, and challenges for integrating health services with climate mitigation targets. This knowledge is crucial to designing effective interventions that can expedite the shift toward a climate-sensitive healthcare system in India.

## Methods

We conducted a narrative review of both peer-reviewed literature and relevant policy documents to map India’s national efforts on healthcare decarbonization and sustainability. The scope was defined to include major initiatives from approximately 2018 onwards, coinciding with the launch of dedicated climate-health policies in India. Our primary aims were to identify: (1) national policies or action plans that address climate change mitigation in the healthcare sector; (2) operational frameworks, guidelines, or standards developed to promote low-carbon and sustainable practices in healthcare facilities; and (3) specific decarbonization strategies being implemented or piloted in the Indian health system (such as renewable energy installations, energy efficiency programs, sustainable hospital accreditation, etc.).

### Search strategy

We searched PubMed and Google Scholar for English-language articles published between 2018 and 2025 using keywords such as *India, healthcare, climate change, carbon footprint, decarbonization, sustainable hospitals, green healthcare, climate-resilient health systems*. We included journal articles, reviews, and viewpoints that discussed India’s healthcare sector in the context of climate change mitigation or sustainability. To capture official initiatives and gray literature, we also reviewed government and institutional websites (Ministry of Health & Family Welfare [MoHFW], National Centre for Disease Control [NCDC], National Accreditation Board for Hospitals & Healthcare Providers [NABH], etc.) for policy briefs, guidelines, and reports (e.g., NAPCCHH document, NPCCHH program descriptions, green hospital guidelines). Bibliographies of key articles were hand-searched to find additional sources.

### Inclusion criteria

We included sources that provided information on policies or interventions aiming to reduce GHG emissions or the environmental impacts of healthcare in India. Global references were included selectively to provide context or recommended strategies relevant to India’s situation. We gave priority to recent sources (2018–2025) and to publications in PubMed-indexed journals when available, to ensure reliability and academic rigor.

### Data extraction

From each source, we extracted details on the policy or strategy described, year of initiation, main objectives, and any reported outcomes or challenges. For national programs (like NPCCHH), we noted their scope and components. For guidelines and standards, we recorded the key domains of action (energy, water, waste, etc.). For implementation examples (such as solar-powered facilities or hospital case studies), we noted the impacts on emissions, cost, or healthcare delivery.

### Synthesis

We abstracted findings thematically and categorized those into five groups: (1) national policy framework; (2) interventions involving greening health infrastructure (building or facility level); (3) renewable energy and/or energy efficiency interventions; (4) other types of decarbonization intervention categories, including transportation supply chain and waste management; and (5) challenges/opportunities for implementation. A table of the main national strategies was compiled, selecting some highlights and features in a clear way. For the same reason, we did not calculate quantitative meta-analysis, but triangulated data from different sources so that findings were broader and more balanced. The findings were contextualized and reviewed through a public health lens, emphasizing how these decarbonization activities converge with health system enhancement and climate goals.

## Discussion

### National policy framework for climate and health

India’s overarching policy response to climate change began incorporating health about a decade ago, setting the stage for current decarbonization efforts in healthcare. The NAPCCHH, published in 2018, was the first dedicated national roadmap at the climate–health interface. It outlined strategies to *“strengthen healthcare services against the adverse impacts of climate change”* and to reduce the health sector’s environmental impact. The NAPCCHH recognized a broad range of climate-sensitive health issues—from heat stress and air pollution to vector-borne diseases and water-borne illnesses—and called for integrating climate adaptation and mitigation into health planning [10]. Building on this, the Ministry of Health and Family Welfare approved the NPCCHH in 2019 under the National Health [11]. The NPCCHH is essentially the implementation arm of the action plan. It focuses on three key pillars: (1) expanding surveillance and public health response for climate-sensitive diseases (with initial focus on heat-related illness and air pollution-related respiratory disease); (2) building capacity and awareness regarding climate and health; and (3) promoting green, climate-resilient healthcare facilities across the country. Notably, heat-wave early warning systems and city/state heat action plans have been developed in several states under NPCCHH’s impetus and have respiratory illness surveillance linked to air quality. These efforts address the *health adaptation* side of climate change. In parallel, NPCCHH explicitly mandates reducing the carbon footprint of healthcare delivery—marking a policy acknowledgment that the health sector must also mitigate its own climate impact [12].

Importantly, India’s climate-health initiatives are situated within broader climate policy and international commitments. While India did not formally pledge a net-zero healthcare target in the COP26 Health Programme (the global initiative for sustainable low-carbon health systems), its domestic policies like NAPCCHH/NPCCHH demonstrate a home-grown commitment to link health sector development with climate action [13]. The inclusion of a Health Mission in the National Climate Change Action Plan since 2015 reflects high-level acknowledgment that health must be part of climate solutions. Additionally, the country’s updated nationally determined contributions (NDCs) emphasize sustainable development co-benefits—implicitly encouraging sectors like health to pursue low-carbon pathways. In 2019, India also became a signatory to the World Health Organization’s initiative on climate-resilient health systems, reinforcing political will to invest in resilient, sustainable healthcare infrastructure. Together, these policies and commitments create an enabling environment for operational measures to decarbonize healthcare [14].

### Greening healthcare infrastructure and operations

A centerpiece of India’s strategy is the push for green and climate-resilient healthcare facilities. This concept encompasses hospitals and clinics that are designed or retrofitted to minimize environmental impact (mitigation) and to withstand climate-related shocks (adaptation). Under the NPCCHH, the NCDC has developed detailed technical guidance for greening healthcare infrastructure. In 2023, NCDC released the *Guidelines for Green and Climate-Resilient Healthcare Facilities*, which specify practical measures for hospitals to become more sustainable [15]. The guidelines cover a comprehensive range of domains: improving energy efficiency (for example, by using energy-efficient lighting, HVAC systems, and medical equipment); conserving water (through rainwater harvesting, recycling, and low-flow fixtures); ensuring buildings are designed for thermal comfort and fire safety even under extreme weather; using eco-friendly construction materials and technologies; enhancing natural lighting and ventilation; and strengthening waste management (both biomedical and general waste) with an emphasis on recycling and safe disposal. By following these measures, hospitals can reduce energy consumption, cut waste generation, and lower their carbon emissions while also creating safer and more resilient healing environments, as mentioned in a recent systematic review [16].

To translate these guidelines into action, pilot projects have been initiated. Some public hospitals are undergoing “green hospital” upgrades in collaboration with the government and technical agencies. For instance, a national hospital energy survey in recent years identified enormous opportunities for efficiency improvements, finding that Indian hospitals, on average, use 50% more energy per bed than global best practices—a gap that can be narrowed with better designs and equipment. In response, hospital administrators in certain states have begun adopting low-cost interventions like LED lighting, solar water heating, and improved insulation to reduce electricity use [17]. The Indian Public Health Standards (IPHS) for primary health centers and district hospitals were updated in 2022 to incorporate environmental sustainability criteria, ensuring that new or upgraded health facilities include features, such as renewable energy backup, proper biomedical waste treatment, and hazard-resistant construction. Moreover, India’s *Kayakalp* initiative—which, since 2015, awards public health facilities for excellence in cleanliness and hygiene—has indirectly spurred environmental improvements (like better waste segregation, sanitation, and infection control). While Kayakalp is not explicitly a climate program, it complements decarbonization by promoting a culture of environmental consciousness in hospitals [18, 19].

Another lever of change is coming from the healthcare quality and accreditation domain. The NABH, India’s apex hospital accreditation body, has recently incorporated sustainability into its assessment standards [20]. In 2024, NABH introduced a Climate Action and Sustainability checklist for hospitals as a voluntary module, aligning with international frameworks of green hospital certification. This includes criteria such as energy management plans, carbon footprint monitoring, sustainable procurement policies (e.g., phasing out ozone-depleting anesthetic gases), and community outreach on environmental health. Hospitals seeking accreditation are now encouraged to adopt these practices, and some leading institutions have begun reporting their carbon reduction measures in quality improvement reports. Such integration of climate-friendly practices into accreditation and licensing can be a powerful driver for widespread adoption, considering the prestige and incentives tied to NABH accreditation [21] (Table 1).

**Table 1:** Key national policies, programs, and operational frameworks supporting decarbonization and climate resilience in India’s healthcare sector.

YEAR	POLICY/PROGRAM	IMPLEMENTING AUTHORITY	CORE FOCUS	RELEVANCE TO HEALTHCARE DECARBONIZATION
2008	National Action Plan on Climate Change (NAPCC)	Government of India	National climate mitigation and adaptation strategy across sectors	Provided the policy foundation for integrating health into climate action and enabled subsequent development of climate-health initiatives
2018	National Action Plan on Climate Change and Human Health (NAPCCHH)	Ministry of Health & Family Welfare/National Centre for Disease Control	Strategic framework addressing climate-sensitive health risks, adaptation, capacity building, and research	Established the national roadmap linking climate change, public health, and sustainable healthcare systems
2019	National Programme on Climate Change and Human Health (NPCCHH)	Ministry of Health & Family Welfare/National Centre for Disease Control (under National Health Mission)	Program implementation focusing on heat-related illness, air pollution surveillance, capacity building, and promotion of green, climate-resilient healthcare facilities	Operationalized climate-health policies and explicitly incorporated low-carbon and climate-resilient healthcare delivery
2021	WHO COP26 Health Programme Commitments	Ministry of Health & Family Welfare (signatory)	Global commitment to climate-resilient and low-carbon health systems	Signaled national intent to align India’s health sector with international low-carbon healthcare goals
2023	Guidelines for Green and Climate-Resilient Healthcare Facilities	Ministry of Health & Family Welfare/National Centre for Disease Control	Energy efficiency, renewable energy, water conservation, waste management, and climate-resilient facility design	Provided technical guidance for reducing healthcare facility-level emissions and improving resilience
2023–2024	NABH Climate Action and Sustainability Initiatives	National Accreditation Board for Hospitals & Healthcare Providers (Quality Council of India)	Incorporation of sustainability metrics into hospital accreditation standards	Incentivized hospitals to adopt energy management, waste reduction, and sustainable procurement practices
2018–present	Solarization of Health Facilities (multiple state and national initiatives)	Ministry of Health & Family Welfare; State Health Departments; Renewable Energy Agencies	Deployment of solar photovoltaic systems in health facilities, particularly primary health centers	

### Renewable energy and energy efficiency initiatives

Transitioning away from fossil fuel-based energy is at the heart of healthcare decarbonization. Indian healthcare facilities, especially large hospitals, are major consumers of electricity, historically drawn from a coal-dominated grid. To address this, solar energy deployment in health facilities has accelerated in recent years. The government, often in partnership with NGOs and international agencies, has launched programs to solarize health centers, particularly in rural and off-grid areas [22]. Notably, the Ministry of Health’s NPCCHH developed *Guidelines for Solar Powering Healthcare Facilities* in 2023, providing a template for states to install and maintain solar photovoltaic systems at hospitals and clinics. By harnessing abundant sunlight, solar panels can supply clean power for lighting, refrigeration, and medical equipment, reducing reliance on diesel generators and grid electricity. This not only cuts carbon emissions but also improves health service reliability—a critical co-benefit for facilities plagued by power outages [23].

Evidence from pilot projects indicates substantial gains. For example, a project to solarize primary health centers (PHCs) in the state of Karnataka demonstrated improved healthcare delivery alongside emission reductions. After installing rooftop solar systems with battery backup, these PHCs experienced uninterrupted electricity supply, enabling them to provide 24 × 7 services even during grid failures. Health workers reported that essential functions like childbirth and emergency care became safer and more efficient since lights and lifesaving devices could run without interruption. Reliable power from solar energy also ensured vaccine refrigerators maintained cold-chain temperatures, and facilities saved significantly on electricity bills. The estimated carbon reductions were notable: each solarized PHC cut its diesel generator use to near-zero, eliminating several tons of CO₂ emissions annually while also reducing local air pollution [24]. Following such successes, solar power systems are being rolled out in numerous health facilities across states, like Chhattisgarh, Assam, and Tamil Nadu, often supported by the Indian Renewable Energy Development Agency and developmental partners. India’s aspiration of *“energy-efficient, solar-driven healthcare”* not only aligns with its renewable energy targets but also builds climate resilience, as solar-powered health centers can keep functioning during extreme weather events that disrupt conventional power [25].

In tandem with renewables, energy efficiency measures are gaining traction. The Bureau of Energy Efficiency (BEE) has included hospitals as a category in its Energy Conservation Building Code and is exploring a star-rating system for hospital energy performance [26]. Some tertiary hospitals have retrofitted old infrastructure with modern, efficient systems: for instance, installing LED lighting, motion-sensor controls, high-efficiency HVAC (heating, ventilation, air conditioning) systems, and building automation to optimize energy use. There is also growing awareness about the climate impact of certain medical practices; for example, the use of desflurane and nitrous oxide in anesthesia, which are potent greenhouse gases. A number of Indian hospitals have joined the global initiative to eliminate high-GWP anesthetic agents or to install gas capture technologies in operating rooms. By switching to alternatives (like sevoflurane or total intravenous anesthesia) and improving anesthetic gas capture, these hospitals significantly cut their GHG emissions without compromising patient care, as reported in pilot studies in Bengaluru and Mumbai [27].

### Sustainable healthcare operations and supply chain

Beyond energy, decarbonizing healthcare requires rethinking many aspects of operations and the supply chain. Transportation is one area of focus: hospital vehicle fleets (ambulances, cold-chain vehicles) and staff travel contribute to emissions. In response, some hospitals are transitioning to electric ambulances and deploying telemedicine to reduce the need for patient travel. During the COVID-19 pandemic, India saw a surge in tele-health consultations, which incidentally has a climate benefit by cutting down on transportation-related emissions [28]. The government’s new *Ayushman Bharat Health and Wellness Centres* (which bring primary care closer to communities) also reduce travel distances for patients, indirectly lowering the carbon footprint of seeking care [29].

The healthcare supply chain, production, procurement, and disposal of medical goods, accounts for a large share of emissions (studies globally estimate 60–70% of healthcare’s footprint comes from the supply chain). In India, efforts are underway to integrate sustainability criteria in public procurement for health. The Ministry of Health has started encouraging procurement of energy-efficient medical devices (like star-rated refrigerators, and efficient sterilizers) and phasing down mercury and other hazardous materials. Furthermore, there is a push to work with the pharmaceutical and medical device industries to adopt greener manufacturing [30]. For instance, the Indian Pharmaceutical Alliance has discussed cleaner production techniques and reducing waste in pharma supply chains, in line with the country’s broader drive for a low-carbon industry. While these supply-chain reforms are at an early stage, they represent an essential frontier; the upstream emissions from the manufacture of drugs, gloves, syringes, equipment, etc., are significant, and greener procurement policies can incentivize manufacturers to reduce their environmental impact [31].

Waste management and circular practices also play a role in decarbonization. Proper biomedical waste management (segregation, autoclaving, safe disposal) is more about infection control and environmental protection, but it can mitigate emissions by avoiding uncontrolled burning of waste. India updated its Biomedical Waste Rules in 2016 and further amendments in 2018, pushing for cleaner disposal technologies and waste minimization [32]. Some hospitals have adopted recycling programs for certain materials, and there are pilot programs for reusing sterilized medical equipment to reduce one-time-use plastics. Additionally, composting of food waste from hospital kitchens and use of biogas digesters for organic waste are being tried in a few eco-friendly hospitals; these reduce methane emissions that would occur if waste went to landfill [33].

A noteworthy development is the growing network of hospitals in India joining the international Global Green and Healthy Hospitals (GGHH) platform. This network provides tools and support for hospitals to track and reduce their environmental footprint [34]. Several large private hospital chains in India (and some government medical college hospitals) have committed to carbon reduction goals as part of their corporate social responsibility and as GGHH members. They report initiatives such as planting trees on campuses, substituting chemicals with green products, and engaging staff in sustainability committees. While these efforts are voluntary and vary widely in scope, they reflect a cultural shift in the healthcare sector toward environmental stewardship [35].

### Challenges and opportunities

India’s journey toward a low-carbon healthcare system, while promising, is not without significant challenges. A foremost issue is implementation capacity and scale. Many of the initiatives so far are at pilot or early stages—for example, a handful of hospitals have achieved notable green upgrades or solar installations, but tens of thousands of facilities nationwide remain untouched. Bridging this gap requires substantial investment and capacity building [36]. The health sector will need increased funding earmarked for climate and environmental upgrades. Currently, the NPCCHH budget is relatively small compared to the vast needs of retrofitting infrastructure across all states. There is an opportunity to integrate health sector climate financing into national climate change budgets and international climate funds. For instance, leveraging India’s partnerships in the International Solar Alliance could help finance solarization of health facilities at scale. Similarly, public–private partnerships may be explored, where private sector expertise in green construction or renewable energy can be harnessed to upgrade government hospitals.

Another challenge is the fragmentation of efforts and the lack of data. Climate-related health programs (like heat illness surveillance) and sustainability initiatives (like energy audits) are often run as separate vertical activities. A more integrated approach is needed, wherein data on hospital energy use, emissions, and climate risks are systematically collected and used to inform health planning. At present, few hospitals in India measure their carbon footprint or track resource consumption in a rigorous way [37]. Incorporating environmental indicators into the HMIS (Health Management Information System) or hospital management systems could enable monitoring progress toward low-carbon goals. Encouragingly, some states are developing Climate and Health Cells within health departments under NPCCHH, which could coordinate data and actions across programs. Academic collaborations can also support evidence generation—for example, conducting research on the health and economic benefits of green hospitals in the Indian context, which can build the business case for wider adoption [36].

Workforce engagement and training represent both a challenge and an opportunity. Healthcare professionals traditionally have not been trained in environmental sustainability. As a result, awareness and technical know-how at the facility level can be limited. Efforts are needed to educate hospital administrators, doctors, and nurses about the importance of decarbonization, not only for planetary health but also for improving patient care and operational efficiency. The inclusion of climate-health topics in medical and public health curricula (as advocated by the planetary health education movement) is a positive step [38]. Moreover, initiatives like hospital “green teams” or appointing a sustainability officer can institutionalize the push for low-carbon operations. Those facilities that have empowered multidisciplinary teams to manage energy, waste, and sustainability have seen quicker progress and staff buy-in [39]. The opportunity lies in reframing sustainability measures as part of quality of care, for instance, emphasizing how improved ventilation and natural light benefit patient recovery, or how reducing pollution improves community health. This can motivate healthcare workers to champion green changes rather than see them as external impositions [40].

There are also broader systemic challenges. Balancing development and emissions reduction is a delicate task for India as a lower-middle-income country. Healthcare access and quality are top priorities; thus, any mitigation measures must be synergistic with improving healthcare delivery, not compromising it. Fortunately, most decarbonization strategies align well with health system improvements, a concept often termed “*smart mitigation.*” For example, energy efficiency saves money that can be reinvested in patient services; renewable energy can ensure more reliable power for rural clinics; telemedicine can expand reach while cutting travel emissions [41]. These co-benefits need to be continually demonstrated to sustain policy momentum. Equity considerations are also paramount: rural and resource-poor health facilities should not be left behind in the greening effort. If anything, they stand to gain resilience (e.g., solar power enabling remote clinics to function off-grid) and should be prioritized. Ensuring that national guidelines are translated into action in all states, including through technical support to lagging regions, will determine how equitable and effective the transition is [25].

Lastly, climate mitigation in healthcare globally is evolving, and India can both learn from and contribute to international experience. The United Kingdom’s NHS, for instance, has pioneered a roadmap to net-zero healthcare by 2040, implementing changes in procurement, building design, and clinical practice. While the contexts differ, Indian policymakers can adapt relevant lessons such as bulk procurement of renewable energy or incentivizing lower carbon clinical pathways. At the same time, India’s innovations, like low-cost solarization models for primary health centers or community engagement for sustainable practices, can serve as examples for other low- and middle-income countries (LMICs) seeking to green their health systems. Collaborative platforms (WHO, Healthcare Without Harm, etc.) and research exchanges can facilitate this two-way learning. As climate change accelerates, the pressure and urgency to decarbonize healthcare will only grow, and staying at the forefront of knowledge will be key.

## Conclusion

India lies at an important convergence of public health and climate change. Climate threats are increasing on one hand, directly affecting the health of millions of Indians, and on the other hand, the healthcare sector’s own environmental footprint is something that cannot be ignored if India has to sustainably develop. This review underscores that India has made a start in earnest for developing a low-carbon health system through some policies (NAPCC-HH, NPCCHH), frameworks (green facility guidelines, solar initiatives), and pilot interventions. These initiatives, which remain in early stages, reflect a recognition that climate mitigation and advances for the health sector really need to go hand in hand. Decarbonizing India’s healthcare is not just an environmental agenda; it is fundamentally a public health opportunity to deliver cleaner, more secure, and resilient care. There is reason for optimism in the findings, while also obvious points of action. Policies in India offers a supportive and changing environment after including hospital considerations mainstreamed in health plans with Indian-specific tools for hospital sustainability. Early adopters from solar-powered rural clinics to tertiary green-accredited hospitals have shown that low-carbon healthcare is possible and beneficial. Patients, too, could stand to gain through cleaner air in a city, hospitals that are better prepared for disasters and potential cost savings passed down with efficiency. But the road before us is long. And transitioning from a handful of success stories to a nationwide transformation will require overcoming financial, technical, and organizational challenges. Effective leadership and governance are critical to guide climate-health actions at different levels of the health system. There should be a greatly increased investment in green infrastructure, perhaps through climate finance. The training of healthcare workers to adopt climate-friendly practices is key to facilitating the uptake of changes at the ground level. So, “Build India’s low-carbon healthcare system” is a lofty goal, yet crucial in the decade ahead. It should put hospital building construction and everyday clinical practice under a sustainability and climate lens. As this review has pointed out, alignment between public health imperatives in India and its climate engagement is not only possible but synergistic. An “all-in- one” climate-smart healthcare system can contribute to the reduction of health-related vulnerabilities while adapting and building an overall greater resilience. For a country as diverse and large as India, success here could be a model for other developing nations. The global public health community, along with policymakers and citizens, must therefore work in harmony to seize the moment and transform hospitals and clinics from contributors to climate change into symbols of solutions that prove another world is possible. India can do so and ensure that the road to health for all in the 21st century is a super highway to planetary health and sustainable future generations.

## Ctri

Not applicable, being a computational study, which is not a clinical trial.
